# Recurrent esophageal stricture from previous caustic ingestion treated with 40-year self-dilation: case report and review of literature

**DOI:** 10.1186/s12876-018-0801-3

**Published:** 2018-05-22

**Authors:** C. Gambardella, A. Allaria, G. Siciliano, C. Mauriello, R. Patrone, N. Avenia, A. Polistena, A. Sanguinetti, S. Napolitano, G. Conzo

**Affiliations:** 1 Department of Cardiothoracic Sciences - University of Campania “Luigi Vanvitelli”, Via Sergio Pansini 5, 80131 Naples, Italy; 2 Endocrine Surgery Unit, University of Perugia, Perugia, Italy; 3 Italian Air Force Medical Corps, Ministry of Defence, Rome, Italy

**Keywords:** Self-dilation, Esophageal stricture, Caustic ingestion, Endoscopic dilatation, Self-bougienage, Case report

## Abstract

**Background:**

Corrosive esophageal strictures are common. The severity of the strictures depends on type, quantity, duration of contact and concentration of the caustic substance ingested. Endoscopic balloon dilation and endoscopic bougienage are a cornerstone in the management of the benign esophageal strictures and are the most widely used treatments, but are expensive and invasive procedures.

**Case Presentation:**

We report the case of an 82-year-old patient with a corrosive esophageal stricture treated for over 40 years by means of home self-bougienage. The procedure has been carried out for the longest lapse of time described in literature, with an excellent control of symptoms. In the case reported, after being carried out for more than 40 years, self-dilation allowed good quality of life and symptoms management, ensuring an excellent nutritional status.

**Conclusions:**

Following an adequate patient training, self-dilatation can be a safe and effective option of treatment, avoiding frequent expensive hospital admissions for endoscopic esophageal dilatation.

## Background

Esophageal caustic ingestions are among the most challenging emergencies for surgeons, otolaryngologists and gastroenterologists [1]. In Italy 15,000 new cases occur annually. They are mainly observed during childhood, and are unintentional in almost all the cases although. Otherwise, in the 90% of observed in adult population, caustic ingestion has a suicidal purpose. Alkaline and acid caustics are the most common chemical substances utilized, causing esophageal burns and producing tissue destruction through coagulation reactions or liquefaction. Acids ingestion causes a coagulative necrosis leading to eschar formation, preventing their action at a greater depth. On the other hand, alkalis ingestion produces liquefactive necrosis generating proteinates and soaps, leading to full thickness lesions [2]. The severity of the lesions depends on type, quantity, duration of contact and concentration of the caustic substance ingested. Acute complications include mucosal injuries, perforations, fistulae, mediastinitis and peritonitis, while long-term complications include esophageal stricture, pyloric stenosis and esophageal squamous cell carcinoma [3–6]. In the management of caustic injuries, one of the crucial aspect is the prevention of esophageal strictures. Endoscopy should be performed within 24 h to evaluate the severity of the lesions and the prognosis. Insertion of a nasogastric tube, antibiotics, early esophageal dilatation and steroids, the latter not routinely used, are among the most important therapeutic weapons to reduce the risk of a severe esophageal stenosis [1]. Surgical management, including esophagectomy, myocutaneous flaps and esophageal replacement procedures, is complex, and carries significant morbidity and mortality [7]. Herein, we describe a rare case of esophageal self-dilatation after caustic soda ingestion by the use of Maloney bougie for the time lapse of about 40 years. We performed a Literature review by a PubMed database search using as keywords “esophageal self-dilation”, “self-bougienage”, “caustic ingestion”, “benign esophageal stenosis”.

## Case presentation

**Fig. 1:**
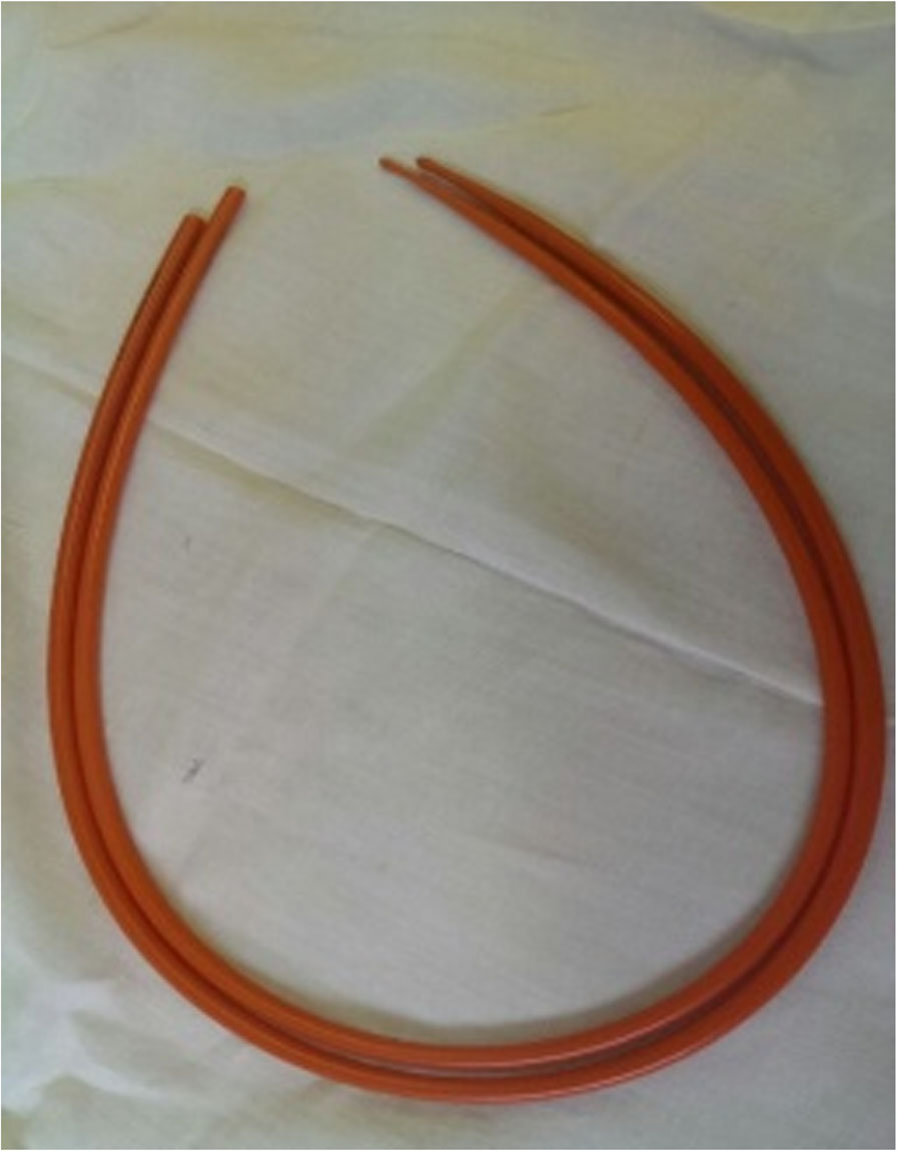
Maloney dilator of 5 mm of calibre

**Fig. 2:**
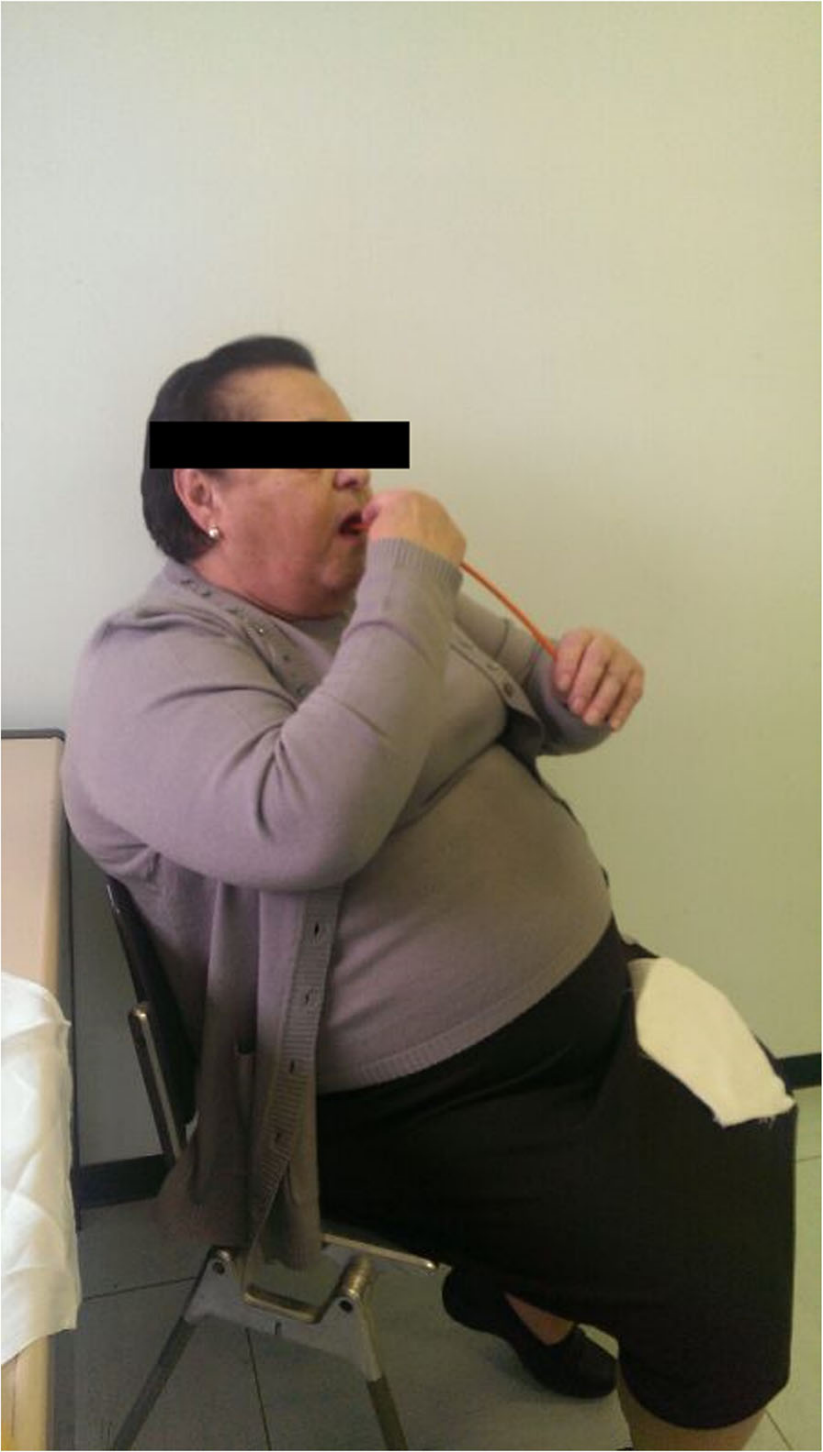
The patient during the self-dilation procedure

**Fig. 3:**
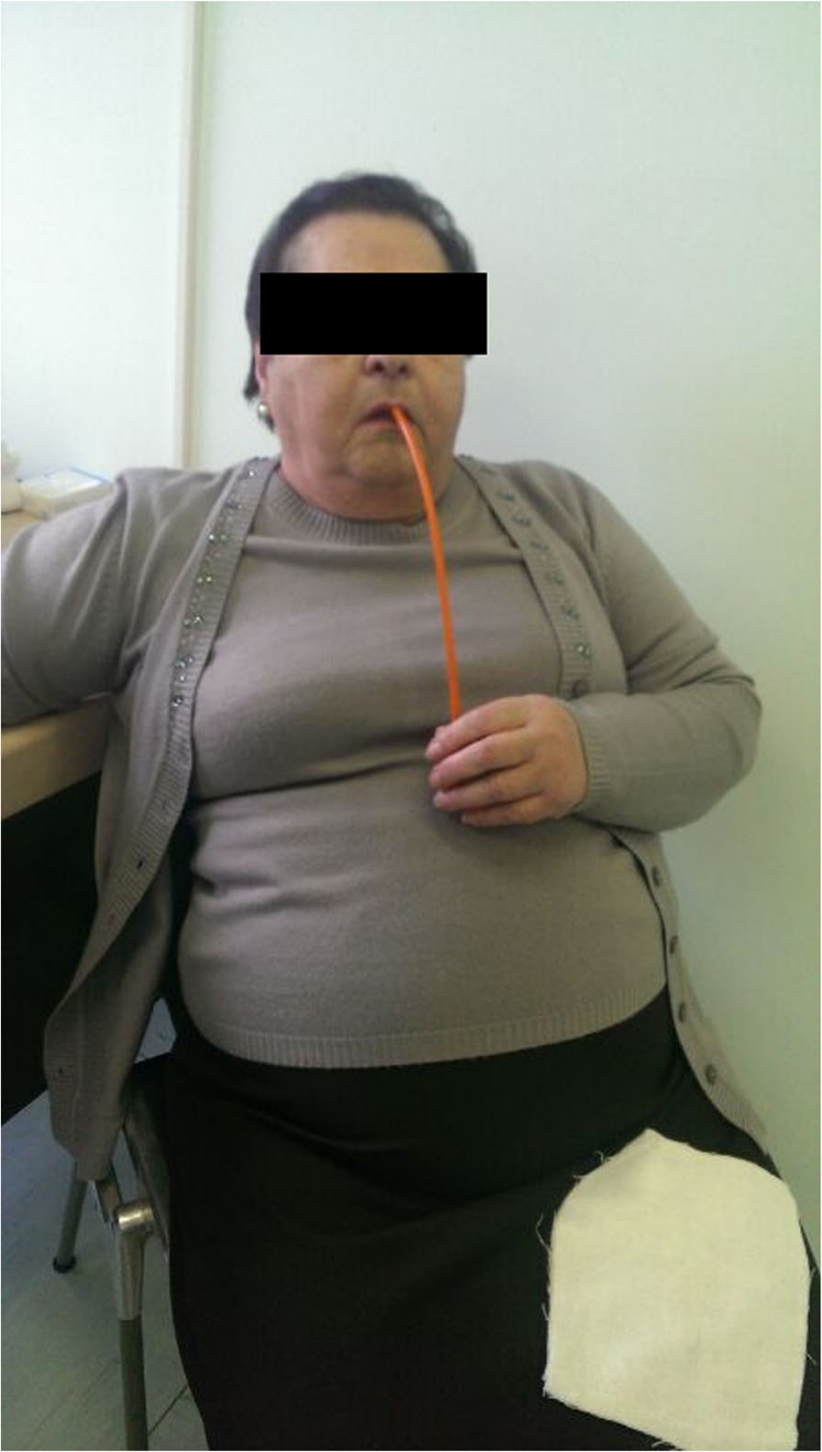
The patient during the self-dilation procedure

**Fig. 4:**
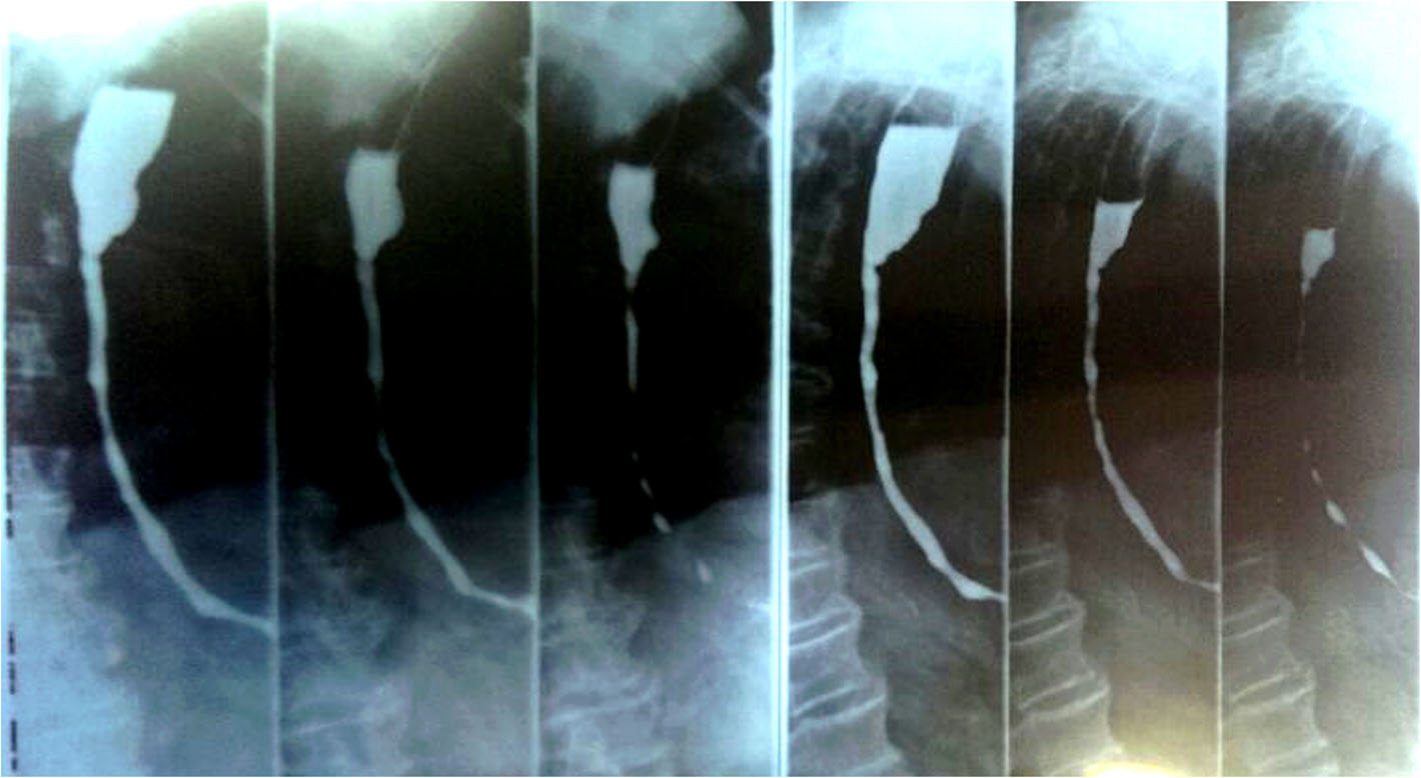
Upper gastrointestinal barium examination showing a stenosis of the medium and lower third of the esophagus with a delayed emptying

In November 2015, we observed a woman 82-year-old, with an episode of caustic soda ingestion at the age of twenty-three for suicidal purpose. At that time, she presented to the emergency department with chest pain, vomit and hematemesis. The patient was hemodynamically stabilized and underwent a gastric lavage. Esophagogastroduodenoscopy showed several esophageal burns, ulcerations and liquefactive necrosis. In the following days, the patient experienced dysphagia, odynophagia, heartburn, hematemesis and weight loss. After three months, the symptoms gradually decreased but a cicatricial fibrotic stenosis of the lower third of the esophagus aroused. The patient started self-dilatation with semi-rigid dilators without reaching any relevant symptomatology relief. At one year from the ingestion, she autonomously bought a Maloney dilator of 5 mm of calibre, and started periodically self-dilatations, after an adequate training, consisting of undergoing physician-performed endoscopic dilation and participating in three self-dilation practice sessions supervised by a physician, according to the technique described by Dzeletovic [8]. The periodical self-dilations and semi-liquid nutrition led to a great improvement of her nutritional status (Figs. 1, 2 and 3). The patient had been in good wellness for about 40 years, when she presented to the Emergency department of Surgical Endoscopy for an esophageal obstruction. A food remnant was endoscopically removed, and for the first time the patient underwent an esophageal dilatation with a Savary bougie. For the following 20 years, the woman has practised occasional Savary bougie endoscopic dilatation (maximum calibre 7 mm) alternated with self-bougienage, achieving a good control of symptoms. Moreover, only over the counter, drugs as dimethicone and sodium alginate have been seldom administered. Only during the last year, the patient has experienced dysphagia, heartburn and nocturnal reflux, so an upper gastrointestinal barium examination has been carried out. A stenosis of the middle and lower third of the oesophagus with a delayed emptying was identified (Fig. 4). An endoscopic 7 mm Savary bougie dilatation was performed with a significantly relief of symptoms. Currently, the patient undergoes monthly standard Savary bougie dilatation with an excellent control of the symptoms.

## Discussion and conclusions

Caustic ingestion for suicidal purpose is not an uncommon event in Western Countries. The overall prevalence of esophageal stenosis is 129 and 122 per 100.000, for men and woman respectively [7]. Endoscopy within the first 24 h has a paramount importance to see the extent of injury and making treatment decisions like conservative vs surgery [2]. The treatment of esophageal stenosis, the most frequent long-term complication of caustic ingestion, is committed to limit dysphagia, avoiding the complications of esophageal obstruction, and preventing recurrence.

Esophageal strictures may be either simple or complex. Complex strictures are often resistant to dilation and are difficult to treat. Generally, they are caused by caustic ingestion, radiation therapy and esophageal anastomosis. Resistant benign esophageal strictures have a negative impact on the quality of life of the patients, and may lead to weight loss, malnutrition and aspiration. Balloon dilatation or endoscopic bougienage are the most widely used treatment, but are expensive and invasive procedures. In fact, endoscopic dilatation requires frequent hospital admissions and multiple sedation procedures, with inherent associated risks and cost, affecting patient productivity and his daily activity [9]. Moreover, despite repeated endoscopic treatment and medical therapy, 30–40% of patients will develop symptoms recurrence within the first year [8]. Patients with recurrent strictures will need repeated monthly or weekly physicians performed dilations, leading to a total dependence of the patient on the medical team. In order to prolong the dysphagia-free period, and to decrease the frequency of repeated dilations compared with conventional endoscopic dilations, intramuscular injections of mitomycin C and steroids were suggested. Mitomycin C is a chemotherapeutic agent derived from some Streptomyces species which reduces scars formation when topically applied to a mucosal lesion, and theoretically should prevents esophageal stenosis in selected cases [10, 11]. Nevertheless, there is evidence that triamcinolone and other steroids injection in combination with endoscopic dilation is able to reduce the risk of recurrent dysphagia in refractory benign esophageal strictures of peptic origin, with results coming from small-sized studies with poorly defined population and not concerning caustic ingestion [12].

A safe, cost saving and useful option in patients who require multiple and frequent dilations to maintain esophageal patency is self-dilation [8, 13]. Home bougienage is generally performed with Maloney dilator of 45 ÷ 60 French, causing only a little discomfort to patients. The best patients for this procedure are those who are self-motivated, compliant, with a normal pharyngeal function and who may be poorly surgically treated. Self-dilatation is cost-effective and safe after a short period of training by experienced surgeons and nurses, consisting of viewing the self-dilation teaching-video, meeting with other patients who perform self-dilation, undergoing physician-performed endoscopic dilation and participating to at least three self-dilation practice sessions supervised by a physician [8–11]. Esophageal perforation, bleeding, bacteremia and aspiration pneumonia, with an overall incidence of 0.3%, are the extremely rare self-dilatation complications [13]. The incidence of perforation, the most dangerous adverse effect, is uncommon and has been reported to be 0.14% [7, 14]. In addition, the inadvertent passage of the dilator into the airways is a rare event that could hesitate in pneumonia or pneumothorax [14].

Few cases of self-bougienage have been previously described in literature, and to our knowledge, the case reported describe the procedure carried out for the longest lapse of time. In fact, for about 40 years the patient has been practicing only the self-dilation, with an excellent symptomatology control, while, following an acute episode of obstruction, occasional Savary bougie endoscopic dilatation alternated with home self-dilation have been performed for other 20 years. Bapat et al., reported a long term symptomatic relief in 51 patients treated with self bougienage for corrosive esophageal strictures as final step of management, with only one case of retraining for procedure failing, after which the patient remained asymptomatic [15]. The same results are reported by Gilmore, Kim and Lee in patients requiring frequent dilatations. [9, 16–18]. Gundogdu et al. reported a higher success rate in patients < 8-year-old, in strictures caused by caustic, in stenosis of the upper third of the esophagus and with stricture length < 5 cm [19]. Dzeletovic et al. reported a more enjoyable life and less interference with daily and social activities for the patients treated with self-dilation, with an improved long-term (32 months) overall health-related quality of life in 90% of cases [7, 13]. Finally, studies from India and China showed favourable results using a Foley catheter and gum elastic bougies [20, 21].

In the case reported, self-dilation, carried out for more than 40 years, allowed a good quality of life and symptoms control ensuring an excellent nutritional status. Even if self-dilation has not received much mention by surgeons and gastroenterologists and remains under-recognized, it can be a safe and effective option of treatment. In selected cases, following an adequate patient instruction and performing periodic instrumental and clinical controls, frequent hospital admissions for endoscopic esophageal dilatation should be avoided, reducing the costs related and favouring a better patient quality of life.
